# The year 2021 in COVID-19 pandemic in children

**DOI:** 10.1186/s13052-022-01360-0

**Published:** 2022-09-05

**Authors:** Elena Bozzola, Carlo Caffarelli, Francesca Santamaria, Giovanni Corsello

**Affiliations:** 1 Department of Pediatric, IRCCS Bambino Gesù Children’s Hospital, Pediatric Diseases Unit, Rome, Italy; 2 Department of Medicine and Surgery, Clinica Pediatrica, Azienda Ospedaliera-Universitaria, University of Parma, Parma, Italy; 3 Department of Translational Medical Sciences, Federico II University, Naples, Italy; 4 Department of Sciences for Health Promotion and Mother and Child Care “G. D’Alessandro”, University of Palermo, Palermo, Italy

**Keywords:** SARS-CoV-2, COVID-19, Children, Prevention, Diagnosis, Transmission, Treatment, Health care facilities, Behaviour

## Abstract

In this article, the developments in the field of COVID-19 pandemic published in the Italian Journal of Pediatrics in 2021 are reflected. We describe progresses in SARS-CoV-2 transmission route, clinical presentation, diagnosis, treatment, and access to health care facilities in children. They led to substantial changes in the clinical approach.

## Introduction

High-profile investigations have been made to characterise coronavirus disease of 2019 (COVID-19) in childhood. Crucial advances in spreading, diagnosis, treatment, and access to health care facilities have been issued in Italian Journal of Pediatrics in 2021. These publications provide important innovations and developments to expand care of children. We focus on the gaps that reports help to clarify through the eyes of key recent studies.

## Methods

For the purpose of the study, reports published in Italian Journal of Pediatrics from May 2020 till December 2021 and concerning COVID 19 pandemics have been examined. Identified key words to perform the research process were: SARS-CoV-2; COVID-19; children; prevention; diagnosis; transmission; treatment; health care facilities; behaviour.

### COVID-19 pandemic among children: transmission and school attendance

In December 2019 a severe acute respiratory syndrome caused by coronavirus 2 (SARS-CoV-2) emerged in China and rapidly spread all over the world, so that on March 2020 the World Health Organization assessed the outbreak as a pandemic emergency [1]. Among European Countries, Italy was the first to be affected by the pandemic and to require restriction, such as lockdown [2]. At the beginning of the emergency, adults, mainly the eldest and the immunodepressed people, were severely affected. Data on the effective burden of the pandemic in children were limited, likely because most were asymptomatic or paucisintomatic. Nevertheless, reports tried to define SARS-CoV-2 infection in children. At the end of the first peak in July 2020, pediatric patients accounted for 1.8% at total confirmed COVID-19 infection pool [3]. Later, after the second peak of pandemic phase, starting from October 2020, reports confirmed an increased susceptibility to SARS-CoV-2 among children. Having an infected cohabitant was strongly associated with the detection of IgG antibodies in children [4].

An interesting and vivid discussion took place on the role of children as carriers of SARS-CoV-2. In this contest, researches tried to discuss the efficacy of school closure for pandemic control [5].

During the first pandemic period, the closure of schools was considered as a measure to control the spread of the virus. In the second one, to prevent and minimize SARS-CoV-2 transmission in the schools, safety procedures were implemented. In details, administrative policies, infrastructural protocols, sanitation, appropriate use of protection devices were the most used measures [6].

To define if the school was a safe place, students and staff members of two schools in Rome were monitored, evaluating the efficacy of prevention measures inside the school buildings. Even if the school has the potential for spreading viruses, preliminary results showed the efficacy of the implementation measurements to contain virus diffusion and not increment the risk of transmission for students [7, 8].

In line with this conclusion, the risk of being positive was five times lower in kids with a school contact rather than those who were household contact [9]. Only few outbreaks were reported in schools, likely to the implemented procedures adopted in classroom after school reopening during the second wave of COVID-19 in Italy [10–12]. Children at school well complied with recommendations for prevention measures such as the use of face masks, hand hygiene and safe distancing [13]. On this base, recommendation was to keep school open, despite COVID 19 pandemic [6, 14, 15].

The role of children in COVID-19 transmission was discussed. Of note, it has been debated if children were at a higher risk than adults to asymptomatically carry the virus in classrooms [3, 16, 17]. An Italian study evidenced that the percentage of asymptomatic SARS-CoV-2 carriers was similar among children and adults after the school reopening in Milan, one of the most involved city in pandemic [18].

### Clinical symptoms

SARS-CoV-2 infection had been associated to a wide spectrum of manifestations ranging from an asymptomatic form to a severe coronavirus case [19, 20].

Among hospitalized children, fever and cough were the most reported symptoms. As for digestive symptoms, they had been less commonly reported [21, 22]. Out of them, vomiting, nausea, diarrhea, and anorexia were the most frequent gastrointestinal symptoms [23, 24]. Of note, the described gastrointestinal manifestation of SARS-CoV-2 infection were non-specific and might mimic other infections, such as acute infective gastroenteritis. This may have led clinicians to underestimate and under-report gastrointestinal symptoms, mainly during the first pandemic period, affecting the overall incidence of digestive symptoms in pediatric COVID-19 patients [25, 26]. Shortness of breath, sore throat, rhinorrhea, conjunctivitis and fatigue were other reported symptoms [27–30]. Nervous system involvement has been seldom reported during COVID-19 or after its recovery [31, 32]. The most reported neurological manifestations were cerebrovascular accidents, reversible splenial lesions, Guillian Barrè Syndrome, benign intracranial hypertension and meningoencephalitis [33–35]. Generally, children affected by COVID-19 neurological manifestations made a complete recovery, although the risk of long-term neurological problems persistence could not be excluded [36, 37].

As for laboratory markers, high values of inflammatory indexes such as C-reactive protein and erythrocyte sedimentation rate were most reported [21].

Of note, it has been recommended to verify if the children were at high risk of a severe form [38–40]. An early identification of infection among at high-risk children was vital to plan medical seek in adequate setting [40]. Either an underlying comorbidity or a heart involvement were mostly observed in severe paediatric cases. Cardiovascular alterations such as reduced left ventricular systolic function with an ejection fraction < 60%, diastolic dysfunction, arrhythmias, including ST segment changes, QTc prolongation and premature atrial or ventricular beat, were identified as the earliest manifestations of heart involvement. Dosing heart enzyme serum levels and studying ventricular function have been found reliable predictive markers for severe clinical manifestations in at risk children [41, 42].

Evidence suggested that symptoms may persist after recovery, as well as in adults [43]. The most reported persistent symptoms were insomnia, fatigue, coughing, dyspnea, loss of taste and/or smell and headaches [43–45]. An Italian study conducted 1–3 months after recovery evidenced that 51% of children still referred at least one symptom, mainly tiredness, loss of taste and/or smell and headaches. The most common post-acute COVID-19 clinical features were noted in 10–18 years old children [46]. Among childhood sequelae associated with COVID-19, multisystem inflammatory syndrome in children (MIS-C) was the most reported. A prompt diagnosis, based on the presence of systemic inflammation and specific organ involvement, was recommended [47, 48].

In details, MIS-C diagnosis should be considered in the presence of a child or adolescent with fever lasting for more than 24 hours plus signs/symptoms of at least 2 organs involvement plus an increased laboratory systemic inflammation indexes (leucocytosis with neutrophilia, erythrocyte sedimentation rate and C-reactive protein (and procalcitonin). The exclusion of infection is required and a recent exposure to SARS-CoV2 may be demonstrated. Anyway, a MIS-C diagnosis should not be delayed by a negative serology. In fact, a prompt treatment is required based on the combined use of corticosteroids, high-dose immunoglobulins and anti-cytokine treatments, depending on the severity of the disease [49, 50].

Evidence suggested that SARS-CoV-2 may act as a trigger capable to determine, in a genetically susceptible individual, a Kawasaki Disease (KD) recurrence. Even if some features of MIS-C overlapped with that of KD, there were epidemiologic and clinical-laboratoristic characteristics that may be useful to differentiate. For example, MIS-C generally occured in older children aged 9–10 years, while KD in those younger than 5 years of age. Coronary artery abnormalities were typical in KD, rarer in MIS-C, associated with higher levels of markers of cardiac injury, like troponin and N-Terminal- B-type natriuretic peptide [51–54].

Few of children affected by MIS-C developed refractory catecholamine-resistant shock along with multi-organ dysfunction, mimicking a toxic shock syndrome, which represented a severe syndrome secondary to uncontrolled activation of the immune system and might be life threatening. Hypotension in these patients resulted from heart failure and the decreased cardiac output [55–58].

Maternal infections during the second- third trimester of pregnancy have been associated with an increased risk of obstetric complications leading to preterm birth, low birth weight and several neonatal diseases, including respiratory distress syndrome, hyperbilirubinemia and need for assistance in Intensive care unit [37].

### Behavior and mental health

Research demonstrated that pandemic and quarantine were psychologically stressful experiences [59]. Mental health status during COVID-19 pandemic outbreaks had been affected mainly among at high-risk categories, such as the youths. Parents as well might have increased youth psychological distress and practiced inappropriate parenting behaviors, which contributed to the development of post-traumatic stress symptoms in children. Becoming more protective, granting less autonomy and communicating a sense of danger might have contributed to the increment of stress symptoms in children [59–62]. Adolescents had to face stress events such as the fear of infection, frustration and boredom related to restrictive measures, lack of face-to-face contact with classmates and friends, loss of relatives. Anxiety and depressive symptoms were the most frequently referred symptoms [63–66]. Children experienced feeling of loneliness and sadness. Sleep disorders increased with irregular sleeping patterns, such as difficulties in falling or staying asleep and increase in nightmares and/or sleep tremors [67, 68]. A likely explanation of these phenomena is that solation might have reduced children’s ability to successfully regulate behavior and emotions and consequently sleep problems emerged or worsened [69, 70]. Evidence supported the need of psychological and psychiatric care for adolescents during the health emergency. Of note, subjects previously affected by psychopathological symptoms were at more risk of psychological impairment than their peers. During the pandemic subjects, especially girls, living in unfavorable environmental context suffered from depression [63].

In this sense, a more frequent smartphone use among Italian children and adolescents during the COVID-19 pandemic, compared to the pre-epidemic period, was observed, likely related to isolation due to restrictive measures. Social media allowed the youth to overcome the stressful period, partially limiting psychological consequences of the lockdown [71–73].

Media devices were useful to allow the youth remaining in contact, to promote learning without interrupting the educational program and to offer psychological and social support [74–77]. Adolescents referred the use of technology for either educational or recreational purposes, demonstrating good levels of resilience [74, 78].

Nevertheless, the use of digital technology for social relationships require a continuous monitoring to prevent an excessive and avoid sleep, emotional and behavioral impairment in children [72, 79–81].

Of note, it has been reported a significant increase of clinical manifestations, including sleep, ocular and musculoskeletal disorders, psychological impairment and social unfavorable outcome, such as a superficial approach to learning and isolation. The risk of overuse and smartphone addiction increased during COVID-19 pandemic [75].

### Treatment

In 2021, evidences rapidly evolved and therapeutic indications changed very quickly in order to either prevent or cure COVID-19 in the best ways [82]. If asymptomatic infection in children did not require a specific treatment, therapeutic options were different on the bases of the presentation. In mild cases, paracetamol in case of fever and airway suction in case of obstruction were indicated, considering monoclonal antibodies only in case of risk factors [83, 84]. In mild forms, medical approach also included oxygen therapy, fluid supplementation, steroids and antivirals [85]. Venous thromboembolism prevention by low molecular-weight heparin was taken in consideration in severe or critical forms [82, 84].

Concerns have been expressed on ibuprofen administration during COVID-19 infection, although no evidence suggested a direct interaction between ibuprofen and ACE2 expression in children [27].

Patients at high risk of developing a severe form might benefit of a specific therapy. For example, treatment with biologics or other conventional immunomodulators should be started and/or regularly continued in inflammatory bowel disease pediatric patients [25]. Nutritional supplements with antimicrobial and immunomodulatory activity were considered as therapeutic adjuvants for the treatment of COVID-19 and for the prevention of viral spreading [86]. In details, vitamin D, probiotics, lactoferrin and zinc were used either to prevent SARSCoV-2 infection or to mitigate the clinical course in infected patients [87–89].

### COVID19 and vaccination strategy

Vaccination is considered the best advisable strategy to prevent COVID-19 infection. Until now, immunization is available for people aged 5 years and older and it demonstrated either a good efficacy or an acceptable security profile [90, 91]. Starting from 2021, in the pediatric age, immunization is actually offered by a Messenger RNA vaccine shot in the muscle of the upper arm. The schedule consists of a 2-dose primary series in children 5–11 years, with a booster dose in case of adolescents. Italian scientific societies recommend COVID-19 vaccination, stating that immunization was also compatible with breastfeeding. In fact, neither evidence nor biological plausibility suggested that vaccine may be dangerous to a breastfed baby. On the other hand, interruption of breastfeeding would led to a certain loss of its well-documented benefits as well as the lack of vaccination would expose the woman to the risk of infection [92, 93].

### COVID 19 and the reduced access to healthcare facilities

Few weeks following COVID-19 pandemic in Italy major changes have been observed in clinical practice at both the hospital and primary care levels. In the early phase of pandemics, in order to contain the infection, several outpatient and inpatient services were discontinued if not considered an emergency [94, 95]. Therefore, outpatient visits to pediatric specialties were reduced by 80%, also due to the parents’ fear of contagious. Later, most activities restarted, but with limitations due to restrictive measures in place. This has led to difficulties in management of chronic conditions with the risk of delaying in the treatment of exacerbations, supplying medical devices, frequency and quality of follow-up visits [94, 96, 97].

The reduction of the access to the first aid and emergency department ranged from 61 to 81% during the first lockdown [98–101]. It correlated with several factors, including a reduced viral exposure due to social distancing measurements and to school-related factors [102–105]. Of note, the greatest decrease concerned accesses for respiratory disorders likely correlated to the widespread use of personal protective equipment as well as to restrictive measures. The very low rate of flu was suggestive of a possible effect played by personal protective equipment, combined with social distancing [98]. Moreover, parents and caregivers preferred to stay at home due to fear of contagious, following the national media campaigns. Therefore, they postponed the access to hospital with the risk of delaying the urgent care for life threating conditions such as appendicitis, diabetic ketoacidosis or neoplastic mass [105].

Telemedicine represented an easy and effective tool during pandemic, reducing the need for patients to attend medical evaluation and guaranteeing the continuity of care [92]. In 2021, the implementation of the use of telemedicine, and the medical teleconsultation partially compensated the reduced ordinary care activities during pandemic [106, 107]. Video consultations and video tutorial or training sessions have been of utility mainly for children with disabilities or complex chronic conditions for provide a regular follow up assessment [106, 108, 109].

Patients with gastrointestinal diseases were often unable to comply with programmed outpatient follow-up visits due to preventive sanitary measures. Telemedicine services have been incremented to guarantee clinical assistance for patients. Nevertheless, several diagnostic procedures were postponed unless they were required as an emergency as gastrointestinal endoscopic procedures were considered at high viral transmission risk. Consequently, the limitation of endoscopic services correlated to the risk of negative outcomes such as delayed diagnoses and psychological distress [25]. As well as medical visit, immunization schedule had been frequently discontinued. An Italian study revealed that approximately 30% of interviewed families skipped vaccination during the first wave of pandemic. In almost half of the cases families referred that they had their children not immunized because they were reluctant to leave home because of misinformation or lack of information on adopted preventive adopted measures or because they fear infection with the COVID-19 virus. Therefore, vaccination coverage had been affected, representing a risk for re-emerge of preventable infectious diseases [110–112].

So, separating well child from ill visits, converting onsite to telemedicine control, performing swab prior to health care access were among the most used tools used to adapt to the pandemic, contrasting the discontinuing pediatric heath care assistance.

### COVID-19 and the rise of obesity and precocious puberty

COVID-19 pandemic and mainly lockdown restriction had negative consequence on life’s style, mainly on sedentary habits [113, 114]. Reduced physical activity as well as an increase of daily meals and junk food consumption correlated with the risk of overweight and obesity among the pediatric population. The level of parents’ instruction played a crucial role, suggesting the importance of promoting the awareness of this health problem among the less instructed people [115, 116]. As well as for obesity, precocious puberty cases in girls incremented since the beginning of the viral pandemic. Researchers speculated whether changes in everyday life might correlate to the finding [117–119].

## Conclusion

Over the past year, research identified main issues of COVID-19 in children that progressed exceptionally quickly. Advances were made in the SARS-CoV-2 transmission, particularly at school. These could be helpful for new prevention strategies. Several studies offered new insights on clinical presentation and sequelae including psychological impairment that will greatly support patient care. Novel options for effective symptomatic treatments were available. Intensive efforts were made to understand which approaches could improve the treatment of pediatric diseases during COVID- 19 pandemic. We anticipate that last year developments ultimately allowed a better management of COVID-19 in childhood.

## Data Availability

Data sharing is not applicable to this article as no datasets were generated or analysed during the current study.

## Abbreviations

COVID-19: Coronavirus disease of 2019
KD: Kawasaki Disease
MIS-C: Multisystem inflammatory syndrome in children
SARS-CoV-2: Severe acute respiratory syndrome coronavirus 2
